# Polyphenol-rich extract of *Saliva chinensis* exhibits anticancer activity in different cancer cell lines, and induces cell cycle arrest at the G0/G1-phase, apoptosis and loss of mitochondrial membrane potential in pancreatic cancer cells

**DOI:** 10.3892/mmr.2021.12101

**Published:** 2021-04-19

**Authors:** Quan Zhao, Xue-Chen Huo, Fu-Dong Sun, Rui-Qian Dong

Mol Med Rep 12: 4843-4850, 2015; DOI: 10.3892/mmr.2015.4074

Following the publication of the above paper, a concerned reader drew to the Editor's attention that several figures (Figs. 3, 4 and 6) contained apparent anomalies, including repeated patternings of data within the same figure panels. Furthermore, Fig. 6 contained data that bore striking similarities to data published in Fig. 8 in another paper published in *Molecular Medicine Reports*, which has now been retracted [Zhu Y-Y, Huang H-Y and Wu Y-L: Anticancer and apoptotic activities of oleanolic acid are mediated through cell cycle arrest and disruption of mitochondrial membrane potential in HepG2 human hepatocellular carcinoma cells. Mol Med Rep 12: 5012-5018, 2015].

After having conducted an independent investigation in the Editorial Office, the Editor of *Molecular Medicine Reports* has determined that the above paper should be retracted from the Journal on account of a lack of confidence concerning the originality and the authenticity of the data. The authors were asked for an explanation to account for these concerns, but the Editorial Office never received any reply. The Editor regrets any inconvenience that has been caused to the readership of the Journal.

